# Using information communication technologies to increase the institutional capacity of local health organisations in Africa: a case study of the Kenya Civil Society Portal for Health

**DOI:** 10.11604/pamj.2015.21.23.5130

**Published:** 2015-05-08

**Authors:** Charles Juma, Aaron Sundsmo, Boniface Maket, Richard Powell, Gilbert Aluoch

**Affiliations:** 1 Danya International, Inc. Nairobi, Kenya

**Keywords:** Africa, capacity building, data for decision making, ICT for development, developing countries, Kenya, knowledge management

## Abstract

**Introduction:**

Achieving the healthcare components of the United Nations’ Millennium Development Goals is significantly premised on effective service delivery by civil society organisations (CSOs). However, many CSOs across Africalack the necessary capacity to perform this role robustly. This paper reports on an evaluation of the use, and perceived impact, of aknowledge management tool upon institutional strengthening among CSOs working in Kenya's health sector.

**Methods:**

Three methods were used: analytics data; user satisfaction surveys; and a furtherkey informant survey.

**Results:**

Satisfaction with the portal was consistently high, with 99% finding the quality and relevance of the content very good or good for institutional strengthening standards, governance, and planning and resource mobilisation. Critical facilitators to the success of knowledge management for CSO institutional strengthening were identified as people/culture (developed resources and organisational narratives) and technology (easily accessible, enabling information exchange, tools/resources available, access to consultants/partners). Critical barriers were identified as people/culture (database limitations, materials limitations, and lack of active users), and process (limited access, limited interactions, and limited approval process).

**Conclusion:**

This pilot study demonstrated the perceived utility of a web-based knowledge management portal among developing nations’ CSOs, with widespread satisfaction across multiple domains, which increased over time. Providing increased opportunities for collective mutual learning, promoting a culture of data use for decision making, and encouraging all health organisations to be learning institutions should be a priority for those interested in promoting sustainable long-term solutions for Africa.

## Introduction

Africa is characterised by a significant burden of communicable and non-communicable diseases, especially in sub-Saharan Africa, the relative distribution of which is projected to shift by 2030 [1]. By 2011, 23.5 million (m) people in the sub-region were living with HIV/AIDS, 69% of the global disease burden [2]. Regionally, cancer is an emerging public health problem [3]. In 2012 there were 645,000 new cases and 456,000 cancer-related deaths in Africa, projected to nearly double (1.28m new cases and 970,000 deaths) by 2030 [4, 5]. Additionally, in 2010 there were 259,500 new cases of, and 2.1m related deaths from, tuberculosis (TB) [6], with the continent accounting for 80% of all TB cases among people living with HIV [7]. Addressing this disease burden, and achieving the healthcare components of the United Nations’ Millennium Development Goals and their planned post-2015 global successors, is significantly premised on the effective delivery of services by indigenous civil society organisations (CSOs). CSOs are an important partner in health care development, performing roles ranging from direct service delivery to advocacy for access to health for all [8]. However, many CSOs across Africa, as in other developing regions, lack the necessary capacity to perform this role robustly. Consequently, implementation constraints arise that include the lack of absorptive capacity, weak organisational governance structures, deficient services’ quality assurance, and human resource limitations. In Kenya, there has been a proliferation in the number of CSOs involved in health- and non-health-related activities - estimated at 8,569 in 2013 [9] - especially in areas not covered by government services. However, a 2012 study by Ekirapa et al, describing the landscape of CSOs in three informal settlements around the country's capital, Nairobi, found that most of the 952 organisations assessed did not possess sufficient capacity to deliver services effectively that would have a demonstrable impact [10].

Measures to address these deficiencies are typically included under the umbrella of capacity building [11]. This traditionally entails the strengthening of systems across multiple functional domains (e.g., financial, human resources, reporting). However, not only can capacity building generally improve service delivery in low-resource settings [12], but, specifically, knowledge management also can be an important contributor to institutional learning and, either directly or indirectly, systems strengthening. Defined as a process of capturing, developing, sharing, and effectively using organisational knowledge [13], knowledge management is a multidisciplined approach to achieving organisational objectives by optimally using knowledge [14]. A discipline used in the fields of business administration, management, and library and information sciences, recently, technology-enabled information systems (e.g., knowledge bases, expert systems, knowledge repositories, group decision support systems, intranets, and computer-supported cooperative networks) have also been used in the pursuit of system-wide organisational objectives by facilitating knowledge sharing as part of a community of practice centred around cross-project exchange and learning. Given a reported 40-60% of knowledge management projects fail [15], research has been conducted to identify the critical factors impacting upon their success. However, not only is variability in operational definitions of such variables undermining comparative analysis, but also the majority of work has been conducted in economically developed nations among large commercial companies and, more recently, small-to-medium enterprises [16]. From a literature review, Karabag [17] identified four critical success factors - management support; motivation; measurement and content quality; and knowledge management system quality - spread across all three knowledge management dimensions: technique, organisation, and humans, with the majority classified under the latter component. These three dimensions have also been classified as people/culture, process, and technology [18].

### Context and FANIKISHA project

Improving the health and well-being of Kenyans is one of the key development areas outlined in the country's *Vision 2030* [19] and the government's *National Health Sector Strategic Plan* [20]. A number of policies and strategies have been articulated to guide health-sector stakeholders in achieving these public health goals, including a community health strategy [21]. This strategy provides government recognition of, and support for, the critical CSO role in delivering social and health services, especially among marginalized, poor, and underserved populations and those in remote and hard-to-reach rural areas. As Kenya endeavours to realize the aspirations of *Vision 2030* and begins to operationalize its 2010 constitution, the health sector faces considerable challenges, including the devolvement of funding and implementation responsibilities for the provision of health and related social services to new county governments. While systems adapt to respond to these new demands, Kenyan public health needs have to be met. Within this challenging and evolving environment, the government relies on CSOs to play a stronger role in responding to patients’ needs, delivering and expanding health services, and forming stronger linkages with other health providers and stakeholders. USAID/Kenya's 5-year Implementation Framework for the Health Sector focuses on supporting local institutions to improve health outcomes and impact through sustainable, country'led programmes and partnerships. The USAID-funded FANIKISHA - which means "accomplish" in Kiswahili - Institutional Strengthening Project, initially scheduled for August 2011-July 2016, aimed to contribute to one of USAID's target results; that is, *"Strengthened leadership, management and governance of health programmes"* by building the capacity of 10 national-level CSOs to play a more strategic advocacy role and strengthening the institutional capacity of their affiliates to provide sustainable leadership for the health and well-being of all Kenyans by: strengthening leadership, management, and governance of local CSOs; increasing access to, and use of, quality data for CSOs’ decision making, and; improving the quality of institutional strengthening for CSOs. The project was implemented by a consortium of partners comprised of Management Sciences for Health, Pact Inc., the Regional AIDS Training Network, and Danya International, the latter taking lead responsibility for the project's information and communications technology (ICT) component: a web-based Kenya Civil Society Portal for Health (KCSPH).

### Evolution of the KCSPH

***Design and development of the national CSO database***: following a mapping exercise of existing web-based resources - which were found not to be comprehensive, contain incomplete information, hosted by multiple agencies, and largely inaccessible or rarely used for decision making on CSO institutional capacity, rendering working and learning together as a community of practice problematic [22] - and an assessment of potential database users’ needs conducted between October 1 - December 31, 2011, the KCSPH was developed to address the identified market gap. This used open-source, non-proprietary software to ensure stakeholders had access to data and the technology to assist with decision-making processes. To ensure full-time support for the portal, the project developed thorough procedural documentation for use by maintenance staff, enabling them to use the software correctly, troubleshoot the system, and adapt it to emerging database users’ needs.

***Design and development of the KCSPH***: the KCSPH, is a comprehensive site that contains the national CSO database, institutional strengthening standards, tools and resources, eLearning modules, geospatial mapping of CSOs’ service delivery areas, and an institutional strengthening market place promoting proven consultants specialising in multiple organisational development areas who could be employed by interested CSOs. The portal's prototype was presented to the following stakeholders, as part of an inclusive, participatory process, requesting input and organisational buy-in: (i) 153 participants from CSOs, development partners, the Government of Kenya, and the private sector; (ii) the FANIKISHA Committee of Advisors; (iii) representatives from the first phase of CSOs during a technical orientation workshop; (iv) the National CSO Database Technical Subcommittee; and (v) the National AIDS Control Council (NACC). Technical leadership of the portal was spearheaded by a Technical Working Group (TWG) consisting of representatives from the government's Ministry of Health (MoH), NACC, Nongovernmental Organisations (NGOs) Co-ordination Board, USAID, Kenya Red Cross, Kenya AIDS NGOs Consortium (KANCO), the African Medical Research Foundation (AMREF), Health NGOs Network (HENNET), another USAID-funded project titled Afya Info, and FANIKISHA consortium partners. The TWG was responsible for the overall technical leadership, direction, and approval of the decisions relating to the design, development, implementation, commissioning, testing, and support of the KCSPH. Following an organisational capacity assessment, it was determined that portal management, following its development, would be the responsibility of the NGOs Co-ordination Board, a State corporation responsible for regulating and enabling the NGO sector in the country. The portal was officially launched in Nairobi in mid-September 2012 [22].

***Features of the KCSPH:*** the consolidated, finalised portal included a number of unique features: a comprehensive CSO database, where CSOs register and provide information on their organisations. Expert staff reviewed organizational submissions for quality, including completeness, accuracy, reliability, timeliness, integrity, and precision; a consultants’ database containing information for vetted consultants in all institutional strengthening categories; a national interactive map that displays the location of CSOs on a geospatial information system (GIS), showing graphically which CSOs and consultants are providing services by location and technical area; online for a facilitating dialogue on various issues relating to institutional strengthening and the CSO sector in Kenya; news and events related to CSOs, institutional strengthening, and health issues in Kenya; eLearning courses/materials for members to enrol and complete the online courses to enhance their knowledge, attitudes, practises, and skills, as well as competencies; and a resource centre that includes institutional strengthening standards, tools, and resource materials. The resource centre includes technical tools developed by other USAID-funded projects, as well as those developed and used by the participating CSOs, and hyperlinks to existing materials, standards, protocols, policies, and guides in multiple health areas (e.g., voluntary care and testing, orphans and vulnerable children, HIV/AIDS prevention, family planning, reproductive health, malaria, TB, and other infectious diseases). Through the FANIKISHA grants, CSO staff could attend technical courses offered and listed in the portal and/or contract-specific tailor-made workshops or technical assistance to build their technical capacity. With these resources and interactive opportunities, the portal served as a forum to exchange information and best practices related to institutional strengthening. A help desk was also established to answer questions and assist users with any web portal or database issues that arose. Lastly, outreach, sensitization, and training activities were conducted with relevant government ministries, USAID-Kenya, donor agencies, and CSOs, providing an overview of the functionality and use of the database, as well as the importance of data for decision making. This paper reports on an exploratory pilot in a non-commercial, economically developing country setting: CSOs working in the health sector in Kenya. The paper reviews the use and perceived impact of a web-based knowledge management portal tool upon the institutional strengthening agenda. Following a description of the context to, and aspects of, the project, the paper outlines the constituent elements of the knowledge portal and its evaluation, identifying factors seen to be enabling or disabling factors in its success.

## Methods

The use and perceived impact of the web health portal was evaluated using three methods: (i) Google analytics data indicating website usage from January 1, 2013 - May 27, 2014; (ii) the comparison of two cross-sectional, online user satisfaction surveys using Survey Monkey, which included eight closed questions, conducted in February 2013 and July 2013, that sought to identify current use of the portal, user needs and ideas for future web enhancements, and a third satisfaction survey covering the period August 2013 - February 2014; (iii) a key informant survey conducted in May 2014 and sent to partnering CSOs’ executive directors, to be completed by those most fully informed to do so (e.g., institutional strengthening leads), and focusing on knowledge sharing and data use to inform organisational decision making.

## Results

### Google analytics data

Over the reference period of 17 months, there were 33,594 logged sessions (monthly average: 1,976), comprised of 19,501 users (monthly average: 1,147), and 145,671 page views (monthly average: 8,568). An average session lasted 6:40 minutes, with an average 4.34 pages accessed per session, and 57.7% of visitors were new to the site, with 42.3% returning visitors.

### User satisfaction surveys

In the first survey the authors received 34 responses from 190 invited registered users (17.9% response rate); in the follow-up survey they received 85 responses from 275 invited users (30.9% response rate). The findings are outlined in Table 1. In the third survey the authors received 176 responses from 700 invited users (25.1% response rate). The findings are outlined in Table 2. Over the reference period, for the first two surveys, the percentage of respondents who found it extremely or very easy to navigate the portal increased from 81-96%, from 85-90% for those who found the information extremely or very clear, from 88-100% who found the portal extremely or very visually appealing, from 69-84% for those who found its content extremely or very uptodate, and from 82-100% for those extremely or very likely to recommend the portal to others, while the percentage of those accessing the portal at least weekly rose from 50-67%. In the third survey, satisfaction with the portal was consistently high, with 98% finding it very useful or useful for their organisation, and individual portal pages ranging from 99% finding it very useful or useful for the CSO database, to 93% for the forum. Similarly, 99% found the quality and relevance of the content very good or good for institutional strengthening standards, governance, and planning and resource mobilisation; 95% for grants and sub-grants. The most common reason for non-registration of a CSO or the creation of its profile was explained by the respondents simply not being individually approached to register (39%). Lastly, 89% found the information contained on the CSO profiles adequate to inform any decision-making process, and 85% reported being satisfied with the adequacy and up-to-date nature of the information contained in the consultant's profile.

**Table 1:** Kenya CSO portal for health user satisfaction surveys (Feb 2013 – Jul 2013)

	Percentage responses	
Question	Feb 2013 (n = 32)	Jul 2013 (n = 85)
1. How easy is it to navigate the Kenya Civil Society Portal for Health (KCSPH)?		
Extremely easy	9	35
Very easy	72	61
Not at all easy	16	3
Did not respond	3	1
2. How easy is it to find information you are looking for on the KCSPH?		
Extremely easy	13	13
Very easy	72	77
Not at all easy	9	10
Did not respond	6	
3. How clear is the information available on the portal?		
Extremely clear	22	20
Very clear	66	73
Not very clear	9	7
Did not respond	3	
4. How visually appealing is the portal?		
Extremely appealing	16	27
Very appealing	72	73
Not at all appealing	9	0
Did not respond	3	
5. How up-to-date is the content on the portal?		
Extremely up to date	0	19
Very up to date	69	65
Not at all up to date	25	16
Did not respond	6	
6. How likely are you to recommend the portal to others?		
Extremely likely	32	58
Very likely	50	42
Not at all likely	9	0
Did not respond	9	
7. How often do you access the portal?		
Daily	12.5	10
Weekly	37.5	57
Monthly	25	27
Very rarely	22	6
Did not respond	3	
8. Would you like to receive emails or SMS updates about new portal content?		
Yes	91	97
No	6	3
Did not respond	3	

**Table 2:** Kenya CSO portal for health user satisfaction survey (Aug 2013 – Feb 2014)

Question	Percentage responses		
	Very useful	Useful	Not useful
1. **Do you find the CSO portal for health useful to your organisation? (n = 176)**	67	31	2
2. **What is your opinion regarding the following CSO portal pages to your organisation? (n = 176)**			
CSO database	68	31	1
Consultants database	44	53	3
Map	44	53	3
Resource centre	63	35	2
E-learning	49	46	5
Links	47	50	3
News & events	53	43	4
Forum	44	49	7
Terms of reference	45	50	5
	**Very good**	**Good**	**Not good**
3. **Rate the quality and relevance of content you found in the different categories on the CSO portal resource centre (n = 176?)**			
Institutional strengthening standards	59	40	1
Governance	55	44	1
Planning & resource mobilisation	55	44	1
Finance	49	48	3
Grants & sub-grants	45	50	5
Advocacy	48	50	2
Communication & IT	53	44	3
HR	45	52	3
M&E	59	38	3
Institutional strengthening	57	39	4
Project management	57	39	4
	**Yes**	**No**	
4. **If your CSO has not registered or created its profile on the CSO portal for health, please indicate why (n = 31)**			
We do not know how to register	32		
We have not been approached to register	39		
We do not have access to the internet	13		
We don't appreciate the value to register	3		
We do not think the portal is relevant to our organisation	7		
Name of the portal has negative connotations for non-CSOs	3		
All of the above	3		
5. **Do you find the information contained on the CSO profiles adequate to inform any decision-making process, especially for donors and implementing partners? (n = 176)**	89	11	
	**Yes**	**No**	
6. **Is the information contained in the consultant's profile adequate and up-to-date? (n = 176)**	85	15	

### Key Informant survey

Out of the 10 CSOs invited to answer the brief survey, 9 responded. Respondents’ perceptions of the effectiveness of the portal at sharing knowledge and at informing decision making within their organisations was unanimously positive. In response to open-ended questions, the main thematic reasons given for this (four for effectiveness of the portal at sharing knowledge, three for its effectiveness at informing organisational decision making), with illustrative quotations, are outlined below (Table 3): access to critical materials, to learning opportunities, to vetted partnering opportunities, and increased organisational visibility were identified as the primary reasons the portal was effective at strengthening knowledge. The practical, consultancy process, and visibility impacts were identified as the primary reasons the portal was effective at informing organisational decision making.

**Table 3:** Thematic reasons for the effectiveness of the portal for sharing knowledge and informing organisational decision making

Why the portal was effective at sharing knowledge	Why the portal was effective at informing organisational decision making
Theme 1: access to critical materials	Theme 1: practical impact
The portal provided materials for institutional strengthening that enabled (our organisation) to review its systems, a case in point in the advocacy materials on coalition building. (#1)	Decisions on skills development through the courses offered, organizations to partner with and consultants available for technical areas in the country. (#2)
I thought it was a good resource and I have picked up tools, and manuals that I will use in my professional practice way beyond the program. The IS Competency Standards will be useful, for now it is clear what it means for a NGO to be a going concern – for an NGO to be of a particular standard. (#9)	Our health department heavily relied on data from CSO portal when developing proposals as well as in making health related decisions. (#8)
**Theme 2: access to learning opportunities**	**Theme 2: consultancy process impact**
The sharing forum allowed (our organisation) to learn about innovative ways of doing business and answered questions that sometimes had bothered the organisation. (#1)	Since the consultants had been pre-qualified by FANIKISHA, we were confident to get the person for the job. All our consultants were gotten from the online portal. (#3)
The web platform was useful in providing information for upcoming events that would interest all the partners present in the portal. (#2)	The information shared on the quality of work done by consultants also informed which consultant to engage. (#5)
It has been effective in terms of information sharing between various organizations, like different events and dates for various CSOs (Sub-grantees). (#5)	It assisted us with marking decisions on consultants. It gave us background information which leads to better decision making. (#9)
**Theme 3: access to vetted partnering opportunities**	**Theme 3: visibility impact**
The fact that the portal was also a point of reference for those looking for organizations to partner with as well as consultants in key technical areas was a good strength of the web portal. (#2)	The portal provided a forum for publicity for (our organisation). Since the most active CSOs were on the home page of the portal, we worked towards being seen and whenever we were not on top, we had to change strategies. (#3)
We were able to understand what other partners are doing across the country. This has resulted in to forging relationships with other FANIKISHA partners who have a similar vision. Were it not for this, it would have been hard to find and work with the partners. (#3)	As an organization we realized for us to engage other actors in our work we must be visible and thus we have been able to make decisions on which partners to work with especially on joint proposals. CSOs that were the most active were given more prominence on the portal by being highlighted on the portal's main homepage and (our organisation) managed to become the most active CSO of the month on the portal and we enjoyed a lot of visibility for that month. (#4)
**Theme 4: access to increased visibility**	
We managed to get positive feedback from other CSOs that we work with closely on our work, which to us is an indication that we have become more visible as a result of the exposure to the portal and that our work was felt widely. (#4)	
Directing traffic to our website, visitors to the CSO portal have been referred to our website increasing the hit rate. (#7)	
The portal has promoted uploads of pictures, videos and short stories for the world at large to go through. (#8)	

Similarly, all thought the portal should be sustained beyond the lifespan of the FANIKISHA project (Table 4). Critical facilitators to the success of knowledge management for CSO institutional strengthening were identified as people/culture (developed resources and organisational narratives) and technology (easily accessible, enabling information exchange, tools/resources available, access to consultants/partners). Critical barriers were identified as people/culture (database limitations, materials limitations, and lack of active users), and process (limited access, limited interactions, and limited approval process).

**Table 4:** Identified facilitators and barriers to the success of knowledge management for CSO institutional strengthening

Critical facilitators	Critical barriers
**(i) People / culture**	**(i) People / culture**
*Developed resources*	*Database limitations*
The training module availed on the portal was a strength that brought in more visitors (#1)	The consultants’ database was not updated as often (#1)
Someone had gone through vast knowledge bases and chosen resources that were relevant and useful for each OCA subject. That makes it a great resource centre. It seemed to me that the resources were practical. (#9)	Not many consultants for the topics required. (#9)
*Organisational narratives*	*Materials limitations*
As source of entertainment in the form of short stories which are knowledgeable to many. (#8)	The resources section lacked materials in some critical categories; material database wasn't updated as often (#1)
CSOs could showcase themselves and their work. (#9)	Have a comprehensive portal where reporting templates could be found. (#3)
	*Lack of active users*
	Lack of motivation to use the portal i.e. the users did not have anything to motivate them to actively use the portal e.g. some social sites use ratings such as likes, stars, comments etc. as a way of pushing users to want to contribute more. (#4)
**(ii) Process**	**(ii) Process**
None	*Limited access*
	It was not accessible to other organizations that do not have internet access (#5)
	*Limited interactions*
	Cap on maximum characters for organizations ‘profile, it didn't include upload of a factsheet, capability statement or photo (#7)
	Inability to take bigger video, that is, when the video exceeds 8mb it cannot be uploaded. (#8)
	*Limited approvals process*
	Posting of TORs or events took approvals, hence time wasting. (#6)
	An organization was not in full control of their account since some posting needed approvals, and very few consultant were found on the portal #6)
**(iii) Technology**	**(iii) Technology**
*Easily accessible*	None
It could be accessed by anyone anywhere (#5)	
Allowed individuals to register and share information (#5)	
*Tools/ resources available*	
Individualised Organizational Assessment tool, which gave instant results (#3)	
Resource Section was very useful. It was a place that was by default for me whenever I needed information on any of the Institutional Strengthening category (#3)	
*Access to consultants / partners*	
Knowing where to get consultants for a certain service made it easy for us to float TORs (#3)	
It was a one-stop shop for consultants (#6)	

## Discussion

This pilot study has a number of limitations, including relatively low survey response rates -which are, however, comparable with many postal and especially online surveys) [23]; a small number of participating CSOs; a non-ranking of the relative importance of those factors identified as facilitators and barriers to the use of knowledge management to strengthen the CSO institutions and their decision-making processes; and the absence of supportive in-depth interview data to explore in detail the issues entailed in the CSOs’ and developing nations’ environmental context. Indeed, future research into this increasingly important field could address these limitations, as well as explore the comparative impact of community of practice membership upon individual and collective experiences within served communities to determine any positive impacts on end users, rather than just upon CSO members.

However, this pilot study has demonstrated the perceived utility of a web-based knowledge management portal among developing nations’ CSOs, with widespread satisfaction across multiple domains, which increased over time as iterative changes in response to users’ preferences were initiated. In general terms the FANIKISHA project has demonstrated a web-based knowledge management portal is both a useful and useable tool for CSO capacity building that can both strengthen institutions and increase the practice of using data for decision making. Moreover, it is interesting that results confirm Karabag's finding that people account for most critical barriers to knowledge management projects, while technology is perceived as a critical facilitator.

## Conclusion

CSOs require technical operational skills and systems to support them; however, these have limited impact if the organisations are unable also to react to changes in their environment through continued learning, among other factors, to inform thought-leadership and decision-making processes that can improve their operations. CSOs ultimately must lead their own improvement processes: scanning their environment, setting priorities, engaging stakeholders, and monitoring and making key decisions that lead to improved programmatic results and ultimately improved patient health outcomes. Knowledge management and encouraging an organisational culture of learning is an integral part of that process. The importance of ICT in the effective implementation of knowledge management initiatives, generally [24], and in healthcare delivery, specifically [25], has been recognised. This pilot study has shown that the KCSPH, as a knowledge management tool, has proven to be an important contributor to institutional capacity building among the CSO community in Kenya. It has increased the reported use of data for decision making among both the MoH and CSOs by providing consolidated, relevant data from a variety of sources in one generally accessible online source and helped build a culture of learning and collaboration across CSOs, government, and the private sector. Providing increased opportunities for collective mutual learning, promoting a culture of using data for decision making, and encouraging all health organisations to be learning institutions should be a priority for those interested in promoting sustainable, long-term solutions for Africa.
